# Monodomain Liquid Crystals of Two-Dimensional Sheets by Boundary-Free Sheargraphy

**DOI:** 10.1007/s40820-022-00925-2

**Published:** 2022-09-19

**Authors:** Min Cao, Senping Liu, Qingli Zhu, Ya Wang, Jingyu Ma, Zeshen Li, Dan Chang, Enhui Zhu, Xin Ming, Florian Puchtler, Josef Breu, Ziliang Wu, Yingjun Liu, Yanqiu Jiang, Zhen Xu, Chao Gao

**Affiliations:** 1 MOE Key Laboratory of Macromolecular Synthesis and Functionalization, Department of Polymer Science and Engineering, Key Laboratory of Adsorption and Separation Materials and Technologies of Zhejiang Province, Zhejiang University, 38 Zheda Road, Hangzhou, 310027 People’s Republic of China; 2 Shanxi-Zheda Institute of Advanced Materials and Chemical Engineering, Taiyuan, People’s Republic of China; 3 State Key Lab of Chemical Engineering, College of Chemical and Biological Engineering, Zhejiang University, 38 Zheda Road, Hangzhou, 310027 People’s Republic of China; 4 Bavarian Polymer Institute and Department of Chemistry, University of Bayreuth, Universitätsstrasse 30, 95440 Bayreuth, Germany

**Keywords:** Monodomain, Liquid crystals, Graphene oxide, Boundary-free sheargraphy, Topological structure

## Abstract

Eliminating topological defects to achieve monodomain liquid crystals is highly significant for the fundamental studies of soft matter and building long-range ordered materials. However, liquid crystals are metastable and sensitive to external stimuli, such as flow, confinement, and electromagnetic fields, which cause their intrinsic polycrystallinity and topological defects. Here, we achieve the monodomain liquid crystals of graphene oxide over 30 cm through boundary-free sheargraphy. The obtained monodomain liquid crystals exhibit large-area uniform alignment of sheets, which has the same optical polarized angle and intensity. The monodomain liquid crystals provide bidirectionally ordered skeletons, which can be applied as lightweight thermal management materials with bidirectionally high thermal and electrical conductivity. Furthermore, we extend the controllable topology of two-dimensional colloids by introducing singularities and disclinations in monodomain liquid crystals. Topological structures with defect strength from − 2 to + 2 were realized. This work provides a facile methodology to study the structural order of soft matter at a macroscopic level, facilitating the fabrication of metamaterials with tunable and highly anisotropic architectures.

**Supplementary Information:**

The online version contains supplementary material available at 10.1007/s40820-022-00925-2.

## Highlights

- Monodomain liquid crystals of graphene oxide over 30 cm is realized by boundary-free sheargraphy.
- The achieved monodomain liquid crystals have bidirectional sheet ordering, exhibiting unique optical, rheological, and conductive properties.
- Boundary-free sheargraphy extends to freely design delicate topology of two-dimensional colloids, including topological structures with defect strength from − 2 to + 2 and complex polydomain.

**Supplementary Information:**

The online version contains supplementary material available at 10.1007/s40820-022-00925-2.

## Graphical abstract

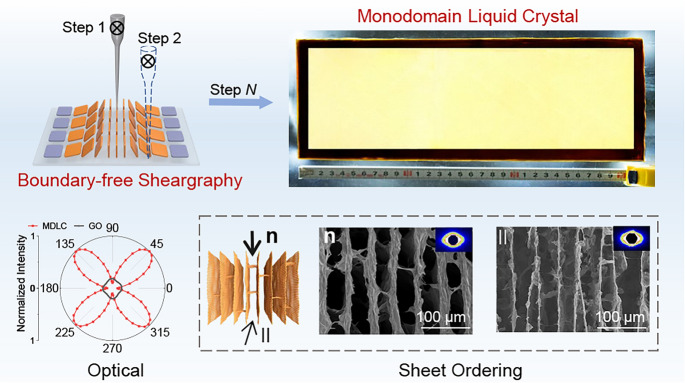

## Introduction

Liquid crystals (LCs), a mesophase with both fluidity of liquids and local ordering like solids [1, 2], have brought about primary scientific revolutions and technological leaps in displays [3], spectroscopy [4], imaging [5], and actuators [6, 7]. Common lyotropic LCs exhibit polycrystallinity and intrinsic defects due to the weak intermolecular interactions of mesogens [2]. Monodomain liquid crystals (MDLCs) are characterized by a long-range monotonic alignment of anisotropic mesogens at macroscopic scales, which endows LCs with advanced functions and anisotropic properties similar to monocrystalline solids [8–10]. However, MDLCs are in a lower entropy state and have stronger elastic stress compared with common LCs, which are prone to relax to the polycrystalline phase. Strict conditions, such as confinement [9, 11], electromagnetic fields [12–15], and surface anchoring [16], have been exploited to manipulate the director field of liquid crystals and retard the relaxation. Currently, the achieved MDLCs of small molecules are only several micrometers thick due to the attenuation of surface anchoring energy or weak confinement effect [17, 18]. MDLCs prepared by electromagnetic fields require continuous external field energy, otherwise they exhibit extremely fast relaxation due to weak mesogen interactions [13]. Achieving macroscopic-scale MDLCs in the free state has not been solved.

Two-dimensional sheets, such as graphene oxide (GO) and hectorite, can spontaneously form lyotropic LCs at low volume fractions due to their giant aspect ratio and good dispersibility [19–21]. The high rotation energy barrier of two-dimensional sheets greatly enlarges the relaxation time of LCs to overcome thermal fluctuation [22]. The slow relaxation attributed to giant molecules underlies the fabrication of monodomain structure in a free state. Meanwhile, two-dimensional sheet ordering is also sensitive to external stimuli because of its fluidity. Electric and magnetic fields that align sheets in isotropic phase fail in nematic phase because of high viscosity and huge energy cost [13, 23, 24]. Shear flow is a valid method to manipulate two-dimensional sheet alignment owing to its mild conditions and manoeuverability. Numerous assemblies of LCs based on shear-induced alignment have been widely reported, such as highly ordered films, fibers, and hydrogels [25–27]. The mechanism is attributed to torque generated by shearing-induced fluctuation in the nematic phase, which reorients two-dimensional sheets in vertical or horizontal alignment to minimize elastic distortion energy [28, 29]. However, shearing fields have intrinsic boundary layers, and the torque decreasing from center to edge of the shearing field renders irregular sheet arrangement, namely π wall defect [30].

Here, we circumvented this intrinsic limitation and produced MDLCs of two-dimensional sheets through boundary-free sheargraphy. The micron-scale shearing field was generated by a programmable moving tip, namely sheargraphy, which reoriented GO sheets and formed a π wall domain. Defects and grain boundaries were eliminated by extremely narrowing the distance between adjacent shearing fields within the width of π wall. The cumulative shearing domain of 2 µm exhibited the uniform shear stress distribution, leading to homeotropic alignment of GO sheets in a large area. The achieved MDLCs exhibited holistic same polarization response, rheological anisotropy, and conductive anisotropy, compared with common GO LCs and π wall defects. The bidirectionally specific thermal and electrical conductivity of MDLC composites exceeded that of most previous nanocomposites, implying the potential usage of MDLCs as lightweight thermal interface materials. Further, controllable topological textures were designed by introducing singularities and disclinations into MDLCs. The MDLCs can be extended to other rich two-dimensional sheets and hold great promise for soft condensed matter with functional architectures.

## Experimental Section

### Materials

Aqueous GO solution (1 wt%, lateral size of 20–50 μm, Fig. S1) was purchased from Hangzhou Gaoxi Technology Co.Ltd. (www.gaoxitech.com). Polydimethylsiloxane (PDMS, Sylgard 184) was supplied by Dow Corning Co., Ltd.. Montmorillonite, molybdenum disulfide, and boron nitride were supplied by Nanjing XFNANO Materials Technology Co., Ltd. Sodium hectorite was prepared by melt synthesis at high temperature followed by a long-term annealing process [31].

### Preparation of GO MDLCs

GO LCs (0.5 wt%) were blade-coated onto a glass substrate to obtain a flat membrane. Then a direct-writing tip, controlled by a robotic arm (C-884 DC Motor Controller, Physik Instrumente, Germany), was immersed into the GO LCs and moved according to programming paths to generate a localized shearing field. The moving accuracy of tip was 1 µm. To generate stable lamellar flow, the diameter and moving speed of the tip were fixed at 26 μm and 3 mm s.^−1^, respectively. Upon shearing, GO LCs formed a *π* wall with a width (2* W*) of around 50 μm (Fig. S4). *D* varied from 200, 50, 25, 10, to 2 µm, and five samples with *D/W* from 8 to 0.08 were prepared (Table S1).

### Fabrication of MDLC Composites

The GO MDLCs were immersed in liquid nitrogen for non-directional freeze-casting process. The frozen GO MDLCs were dried (Pressure: 10 Pa; Temperature: − 80 °C) with a freeze-dryer (LJG 10, Songyuan Freeze Dryer, China) for 48 h. Then dried skeletons were annealed at 3000 °C in Ar atmosphere to get high-quality graphene skeletons. MDLC composites were fabricated by using the vacuum-assisted impregnation method. First, prepolymers (A and B) of PDMS and ethyl acetate were uniformly mixed at room temperature with the weight ratio of 10:1:1. The graphene skeletons of MDLCs were then entirely immersed into the mixture. After infiltration for 1 h, the samples were transferred into a vacuum oven at ambient temperature for 6 h to remove air and finally cured at 100 °C for 1 h.

### Characterization

The birefringence of the GO MDLCs was observed under a polarizing optical microscope (ZEISS Axio Scope. A1). Transmitted intensity was calculated based on the grayscale of the polarized optical microscope (POM) image by using Image J. Small-angle X-ray scattering (SAXS) measurement was taken on a Xeuss SAXS system (Xenocs SA). Scanning electron microscopy (SEM) images were taken on Hitachi SU-8010 field emission system. Rheological measurements were taken at 25 °C with a stress-controlled rheometer Hacke MARS (Thermo Fisher, Germany) using a cone and plate geometry with a diameter of 8 mm and cone angle of 1°. The dynamic frequency scanning was performed at 0.1% strain in the angular frequency range of 0.1–100 rad s^−1^. Anisotropic flow properties of GO MDLCs (1 mm × 15 mm × 15 mm, 1.3 wt%) were measured by using a Keysight T150 UTM. The sample was sandwiched between two pieces of glass, and shearing force was measured by pulling a piece of glass at a constant displacement rate (100 µm s^−1^) in displacement control mode. Electrical conductivity was measured by a standard four-probe method composing a Keithley 2400 multiple-function source-meter [32]. Thermal conductivity was calculated by:

1 $$K = \alpha \times C_{p} \times \rho$$

where *α* is the thermal diffusivity, *C*_p_ is the specific heat capacity, and *ρ* is the density of the sample. The thermal diffusivities were measured by using the laser flash method (LFA 447, NETZSCH Leading Thermal Analysis, No:15010459, Germany). The specific heat capacity was calculated using differential scanning calorimeter (Q200, TA Instrument). The density of samples was calculated by dividing mass by volume.

## Results and Discussion

### Fabrication of MDLCs

During boundary-free sheargraphy process, a movable tip was immersed in GO LCs and submerged to the up surface of stage (Fig. S2a). By moving the tip, a shearing stimulus was generated to reorient GO sheets along the shearing direction, forming a *π* wall with width of 2* W* (Fig. S2b). The width of *π* wall from 48 to 510 μm can be precisely tuned by switching tip diameters or adjusting moving speeds (Fig. S3). From the center to the boundary of *π* wall, GO sheet ordering gradually changed from homeotropic alignment to horizontal alignment (Fig. S2c-f).

**Fig. 1:**
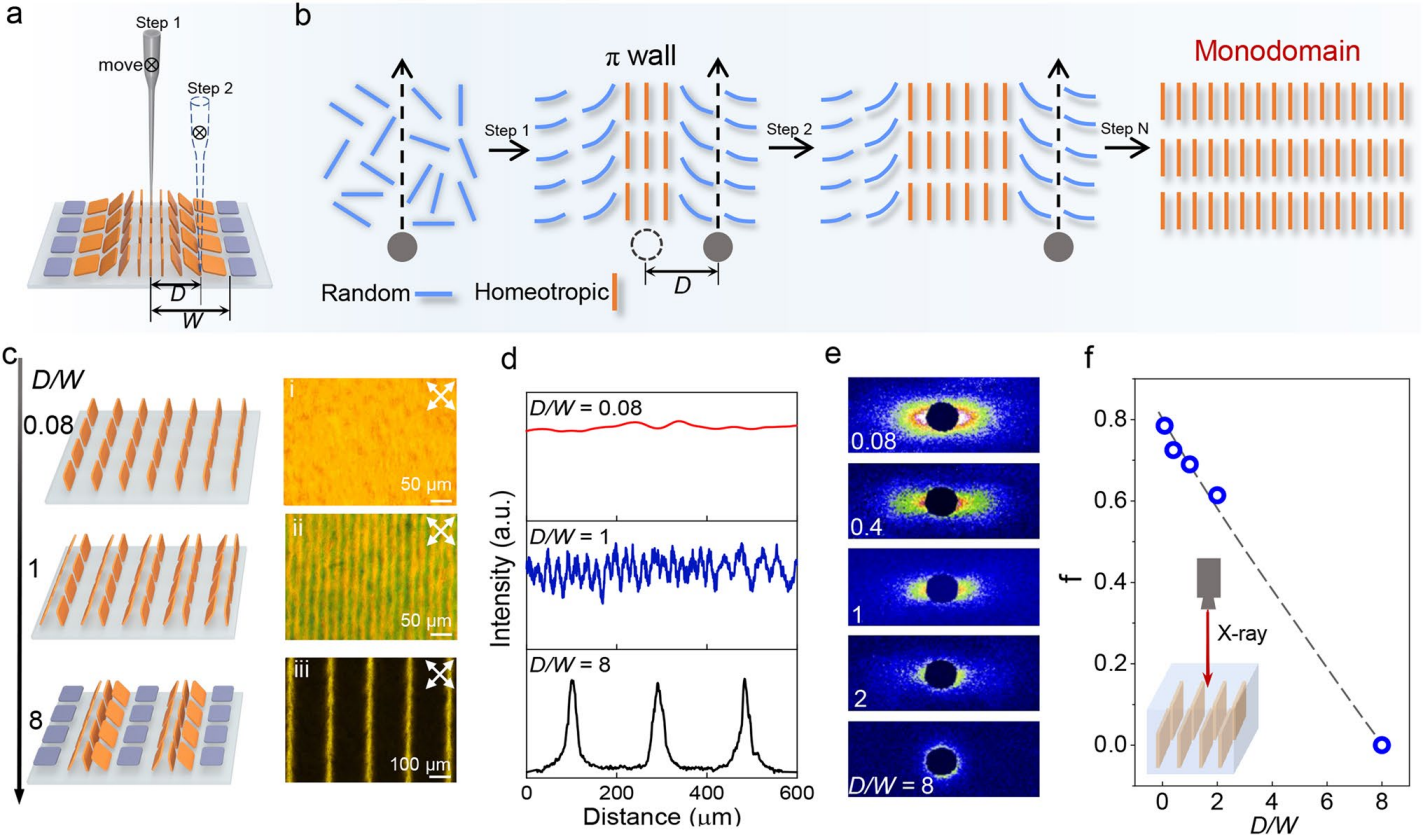
**a** Schematic of boundary-free sheargraphy method. *W* is the half width of a π wall and *D* is the distance between two adjacent localized shearing fields. **b** The fabrication of GO MDLCs by controlling cumulative micron-scale shearing field. Step *N* is the number of cumulative shearing. **c** Three topological architectures of GO LCs as a function of *D/W*, (i) *D/W* = 0.08, (ii) *D/W* = 1, (iii) *D/W* = 8. All POM images were viewed with boundary lines at 45° to the polarizers. **d** Transmission intensity of POM images. **e** SAXS patterns. **f** The orientation order parameter (*f*) as a function of *D/W*

To eliminate π wall and intrinsic topological defects of GO LCs, a boundary-free shearing field was generated by extremely tightening the distance (*D*) between adjacent shearing fields (Fig. 1a–b). During the second shearing, the track of tip (set to 2 µm) was inside the boundary of the first shearing field, eliminating boundary layer of π wall. Shearing stress was uniform and GO sheets were highly oriented in a tiny region of 2 µm. Simultaneously, flow field of the second shearing did not affect the vertically aligned tiny domain generated in the first shearing due to high rotation energy barrier. After *N* times of cumulative shearing, long-distance (*N* $\times$ *D*) monodomain liquid crystals (MDLCs) were obtained through multiple accumulations of highly uniform tiny domain (Fig. 1b).

Topological structures of GO LCs presented manifest variation from random distribution to monodomain configuration by tailoring the *D/W*, where *D* is the distance between adjacent shearing fields and *W* is the half width of π wall domain (Fig. 1c–d and Table S1). Upon shearing, GO LCs formed a *π* wall with a width (2* W*) of 50 μm (Fig. S4). *D* varied from 200, 50, 25, 10, to 2 µm. By tuning the step size of the tip at 200 μm (*D/W* = 8, *D* was much larger than *W*), the shearing region presented discontinuous periodic *π* wall configuration (Fig. 1c-(iii)). The polarized optical microscopy (POM) images and corresponding transmission intensity showed bright and dark domains with a period of 200 µm, indicating the coexistence of *π* wall defects and intrinsic topological defects of GO LCs (Fig. 1d). When *D/W* = 1 (*D* was fixed at 25 µm), the topological architecture consisted of continuous half π wall and its POM image (Fig. 1c-(ii)) displayed a continuous but nonuniform texture. Other structures with *D/W* = 2 and *D/W* = 0.4 also exhibited nonuniform textures due to fluctuant shearing stress (Fig. S5). Further decreasing the *D* to 2 µm (*D/W* = 0.08), whole region formed MDLCs with uniform optical texture (Fig. 1c-(i)). Corresponding transmission intensity of POM image reached a stable maximum value over the entire region (Fig. 1d). SAXS was conducted to prove the high orientation degree of MDLCs quantitatively. When *D/W* was controlled at 0.08, SAXS pattern of GO MDLCs exhibited strong equatorial streak scattering pattern. Calculated order parameter was as high as 0.78 (Figs. 1e–f and S6), illustrating that the GO sheets of MDLCs were both homeotropically oriented and aligned parallel to the shearing direction. SEM images showed distinctive frozen configurations changing from π wall defects to vertical sheet array when gradually narrowing *D* down to 2 µm (Fig. S7). The experimental results above suggested that GO MDLCs were successfully prepared when *D* was greatly less than *W*.

### Optical and Rheological Properties of MDLCs

**Fig. 2:**
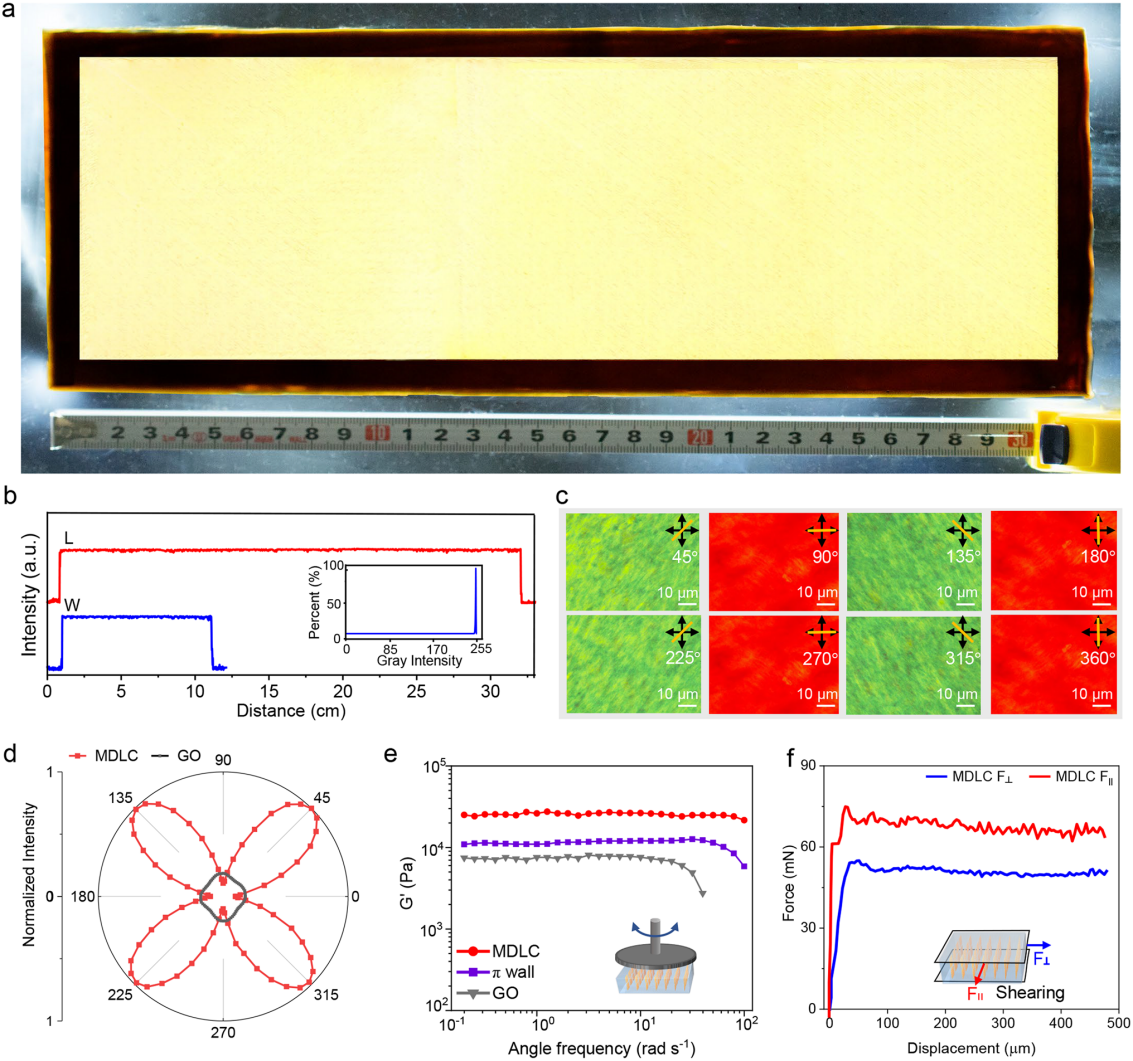
**a** GO MDLCs with the area of 30 cm $\times$ 12 cm. **b** Transmitted intensity of POM images in orthogonal direction. L is the length direction and W is the width direction. The insert is gray distribution in the whole area. **c** Birefringence of GO MDLCs observed between polarizers with a compensation plate of 530 nm by rotating samples from 45° to 360°. **d** Transmitted brightness variation of GO MDLCs and common GO LCs from 0° to 360°. **e** Angular frequency dependence of the elastic modulus (G`). GO MDLCs and π wall defects were prepared with *D/W* = 0.08 and *D/W* = 2, respectively. **f** Anisotropic flow properties of GO MDLCs in the orthogonal direction

Benefiting from high precision and efficiency of the boundary-free sheargraphy method, GO MDLCs with a large area of 30 cm × 12 cm was fabricated (Fig. 2a). Transmitted intensity of GO MDLCs was uniform, and gray value of whole region presented a single distribution near 255 (Fig. 2b). The narrow gray distribution proved that *π* wall defects were dislodged and GO sheets oriented uniformly in boundary-free shearing fields. To identify the orientation direction of GO sheets, we tracked the polarization response of GO MDLCs by rotating the sample from 0° to 360°. Strong birefringence color with the brightest green appeared at a crossing angle of 45° and transmitted intensity also reached the highest value at 45°, both changing periodically bright-dark transition per rotated 90° (Fig. 2c–d). In contrast, transmitted intensity of common GO LCs was 90% lower than that of MDLCs and its polarization response lacked obvious periodicity because of its polycrystallinity feature. Polarization response of GO MDLCs implies that GO sheets are homeotropic and aligned along the shear direction, which is bidirectionally ordered structures (Note S1). Therefore, the highly ordered sheet array endowed the large-area MDLCs with the same birefringence and sensitive polarization response.

GO MDLCs displayed distinctive rheology behavior in shearing flow fields because of the sheet ordering along biaxial directions, compared with common GO LCs. The elastic modulus of MDLCs in oscillation fields was much higher than those of GO LCs with randomly aligned domains and π wall defects (Fig. 2e). Higher elastic modulus is attributed to the regularity of GO sheet array. Highly ordered structure increases the interactions between sheets, like Van der Waals force and inner structure stress, leading to higher ability to resist shear deformation [33]. In addition, MDLCs showed anisotropic flow properties when stimulated by an external shearing field. In Fig. 2f, shearing force (*F*_*‖*_) was 70 mN when the shearing direction was parallel to sheet alignment, 30% larger than $F_{ \bot }$ (shearing force perpendicular to sheet alignment). Common GO LCs showed isotropic flow properties with a shearing force of 30 mN (Fig. S8), indicating random distribution of sheets and low elastic modulus. The difference between the *F*_*‖*_ and $F_{ \bot }$ of the MDLCs corresponds to the anisotropic structure. Highly ordered sheet array parallel to the shearing direction drastically fortifies the interlayer interaction of sheets, resulting in the higher locomotion energy to induce the sheet deformation and flow. Therefore, MDLCs displayed anisotropic rheological properties in biaxial directions. Compared with polycrystalline GO LCs, distinctive rheological properties of MDLCs revealed the uniform long-distance sheet array and anisotropic structure.

### Spatially Regular Skeletons of Dried GO MDLCs

**Fig. 3:**
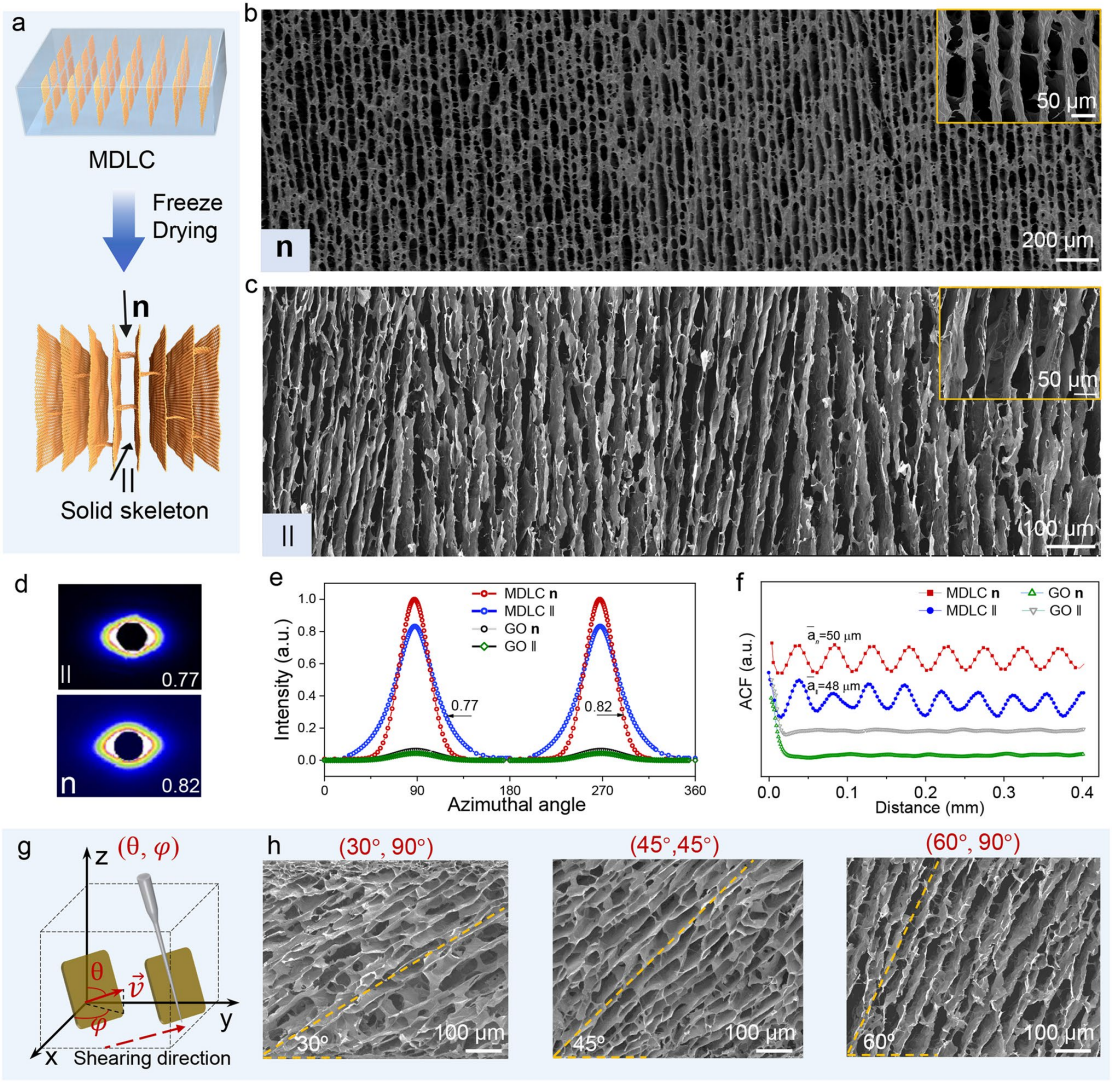
**a** Schematic of dried GO MDLCs by freeze-drying. **b–c** SEM images of solid skeleton from **n** and II directions. The insert images are SEM images with higher magnification. **d** two-dimensional SAXS patterns. **e** Corresponding azimuthal angle plots from **n** and II directions. **f** Autocorrelation functions, suggesting the ensemble average of lamellar spacing from **n** and II directions. **g** Schematic of spatial orientation ($\uptheta$, φ) of tilted MDLCs. θ is the zenithal angle (the angle between the director ($\overrightarrow{v}$) of GO sheet and the z-axis), and is the azimuthal angle. **h** Monodomain skeleton with tilted sheet alignment. All samples were prepared with *D/W* = 0.08

We obtained the regular skeleton of dried GO MDLCs by the freeze-drying method, showing a long-range uniform lamellar structure (Fig. 3a). SEM images from both **n** and II directions demonstrated that the skeleton presented long-distance layered graphene arrays with interval spacing around 50 µm (Fig. 3b–c). The autocorrelation functions in both **n** and II directions were calculated to evaluate structural ordering. The results consisted well with SEM images, reflecting periodic spacing of 50 µm (Fig. 3f). SAXS patterns of monodomain skeleton had two peaks at azimuth angles of 90° and 270° (Fig. 3d–e), corresponding to orientation ordering parameters of 0.82 (**n**) and 0.77 (II), while common GO LCs showed isotropic scattering rings (Fig. S9c).

In addition, by adjusting the spatial angle of the shearing field (Fig. S10a-b), MDLCs with arbitrarily tunable director orientation ($\uptheta$, φ) were realized (Fig. 3g). The tilted MDLCs also displayed uniform texture and sensitive polarization response (Fig. S10c-e). Due to the tilted arrangement of GO sheets, the transmitted intensity gradually decreased with the increased θ (Fig. S10f-g). After freeze-drying process, the monodomain skeleton exhibited various tilted states (0 < θ < 90°, 0 < φ < 90°), including 30°, 45°, and 60° (Fig. 3h). The spatially regular skeletons are inherited from the ordering sheet array in the colloidal state. Conversely, common GO LCs had rich topological defects and exhibited disordering sheet arrangement. During freeze-drying process, the nucleation of ice crystals occurred randomly in the whole sample and GO sheets were easily extruded to the grain boundaries of ice crystals [34, 35], and thus the skeleton of common GO LCs was randomly porous and disordered (Fig. S9).

### Monodomain Structure of Diverse Two-dimensional Sheets

**Fig. 4:**
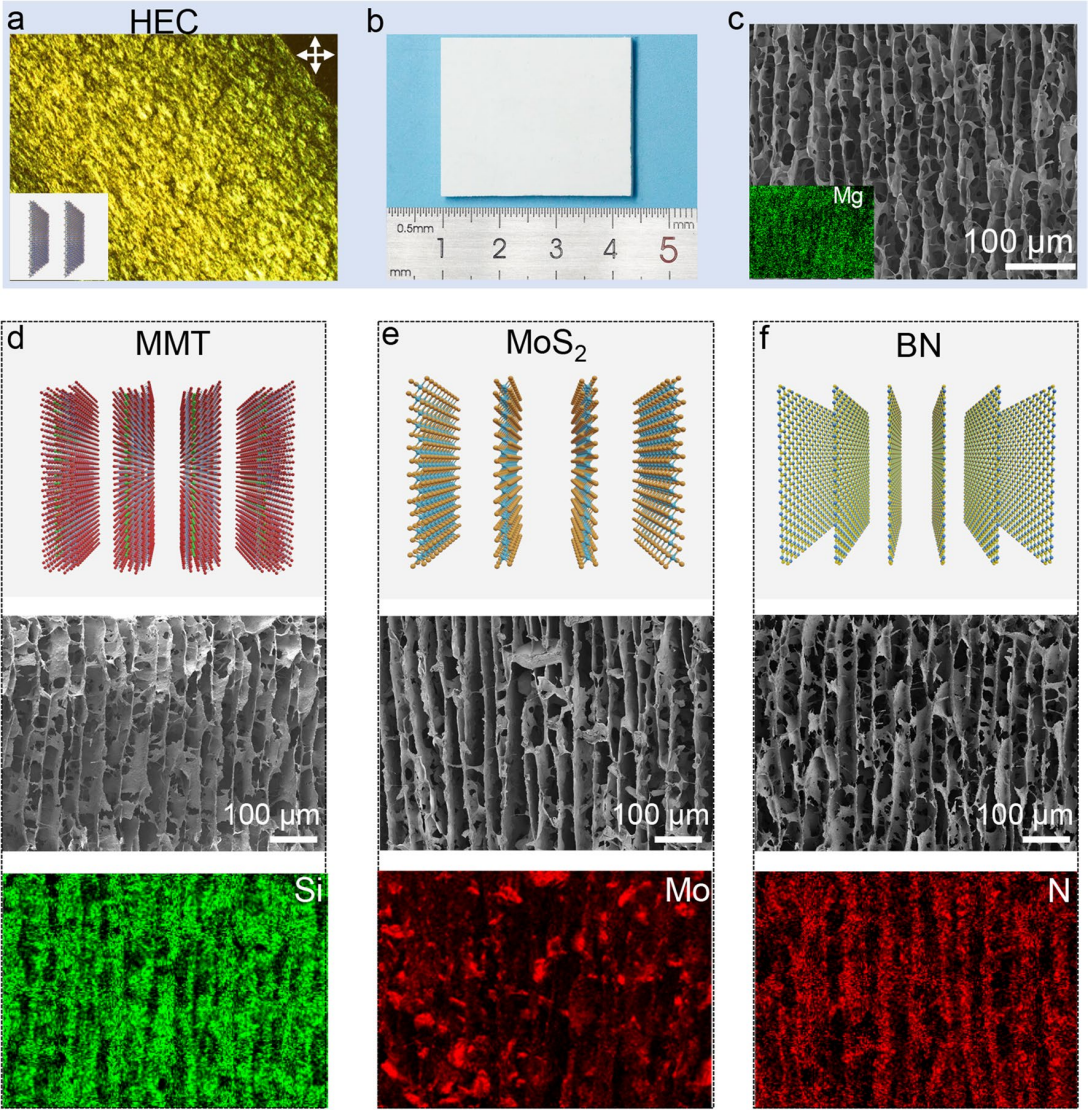
**a** MDLCs of sodium hectorite (HEC). **b** Macroscopic images of dried monodomain skeleton of HEC. **c** Cross-sectional SEM image. Insert is energy-dispersive spectrometer images. **d-f** Schematics of monodomain structures, cross-sectional SEM and energy-dispersive spectrometer images of montmorillonite (MMT), molybdenum disulfide (MoS_2_), and boron nitride (BN), respectively

MDLCs can be extended to a wide range of two-dimensional sheets from minerals (sodium hectorite, montmorillonite), insulator (boron nitride), to semiconductor (molybdenum disulfide) via boundary-free sheargraphy method (Fig. 4). For example, sodium hectorite has a giant aspect ratio of ~ 20,000 with monoatomic thickness and spontaneously forms homogeneous liquid crystals at a content as low as 0.3 wt% due to high charge density [31]. These properties illustrate high rotational energy of sheets and retardation of relaxation. Its nematic phases were aligned into monodomain texture by the boundary-free sheargraphy method, and the achieved MDLCs of sodium hectorite exhibited a homogeneously bright texture at a content of 1.2 wt% (Fig. 4a) and ordered sheet arrays (Fig. 4b–c). Molybdenum disulfide was exfoliated to form homogeneous nematic phase [36] and its monodomain structure was realized by the boundary-free sheargraphy method (Fig. 4e). Contrary to sodium hectorite, the aqueous dispersion of boron nitride and montmorillonite tended to precipitate because of their low solubility and weak interlaminar attractions. To obtain homogeneous dispersions, a small amount of GO was introduced as an additive to retard the relaxation of the two-dimensional sheets. The weight ratio was controlled at 1:70 (GO: boron nitride) and 1:100 (GO: montmorillonite), respectively. The tiny amount of GO can be further eliminated by heat treatment at 500 °C and the regularity of skeleton was well maintained. As shown in Fig. 4d, f, their skeletons exhibited ordered lamellar structures, indicating that monodomain sheet ordering was achieved by boundary-free sheargraphy method in the colloidal state.

### Anisotropic Conductivity of MDLC Composites

**Fig. 5:**
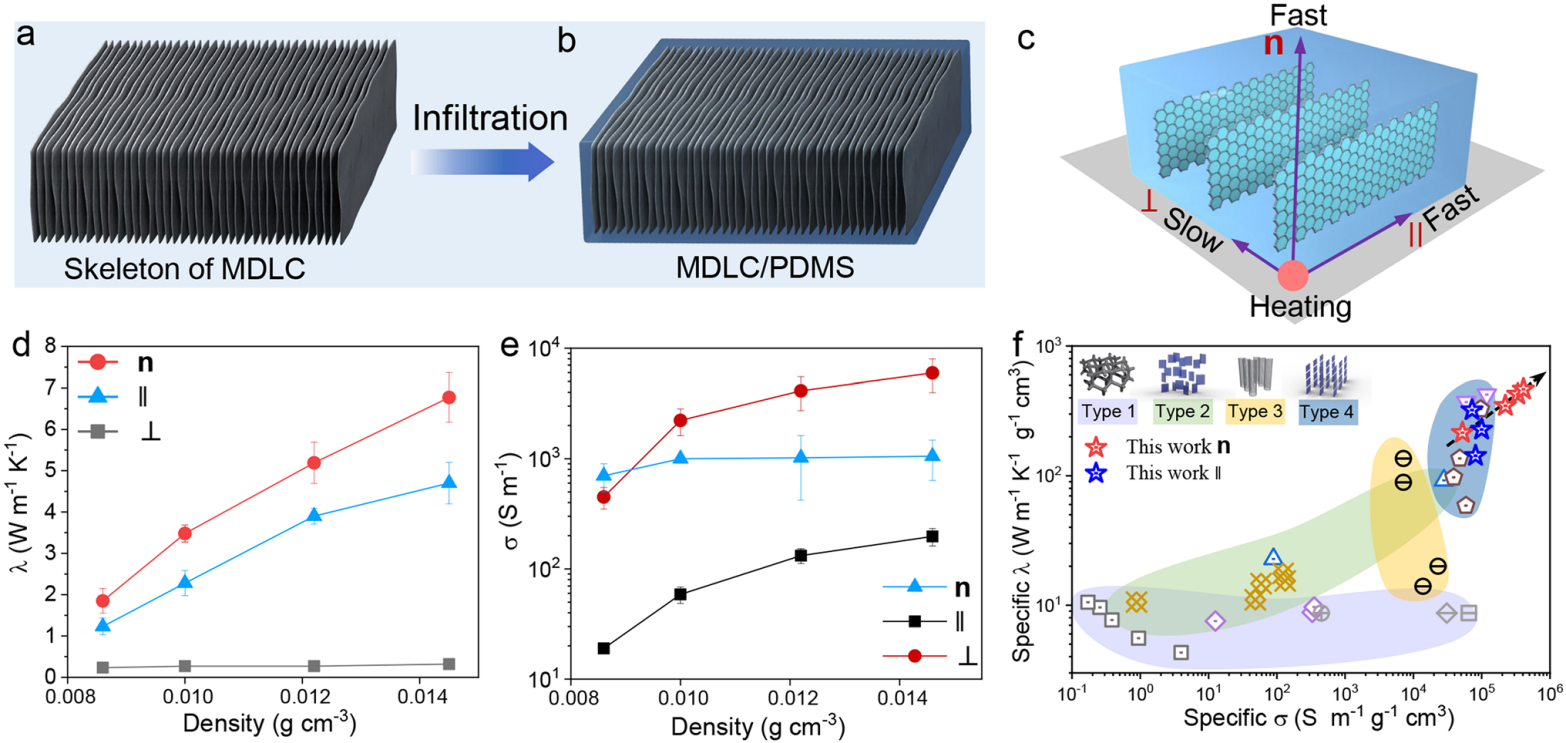
**a** Skeleton of MDLCs with thermal graphitization of 3000 °C. **b** MDLC/ polydimethylsiloxane (PDMS) composites. **c** Schematic diagram of the biaxial thermal conductance. **d** Thermal conductivity (λ) in three directions (**n**, II, ⊥). **e** Electrical conductivity (σ) in three directions (**n**, II, ⊥). **f** A comparison of specific *σ* and specific λ of the MDLC/PDMS composites and other composites with different conductive architectures

We fabricated MDLC/PDMS composites, and the regular skeletons brought about distinctively bidirectional conductivity in the composites. The skeleton of GO MDLCs was first thermally annealed at ~ 3000 °C and then vacuum-infiltrated with PDMS (Fig. 5a-b and S11). The skeleton in composites also showed high lamellar ordering (Fig. S11c). Bidirectional sheet arrangement of MDLCs enhances the transfer of phonon and electron, which endows the composites with outstanding thermal and electrical conductivity in biaxial (**n** and II) directions (Fig. 5c). In Fig. 5d, the thermal conductivity (λ) of MDLC composites in **n** and II directions was 6.77 and 4.71 W m^−1^ K^−1^ (**II**), respectively, which were 21 and 15 times higher than that in ⊥ direction (0.32 W m^−1^ K^−1^). The electrical conductivity (σ) of MDLC composites was measured as high as 5.9 × 10^3^ S m^−1^ (**n** direction) and 1.1 × 10^3^ S m^−1^ (II direction), 29 times and 5 times exceeding the σ (197 S m^−1^) in ⊥ direction (Fig. 5e).

Given the excellent biaxial electrical and thermal conductivity of MDLCs composites at relatively low graphene content, we compared enhancement efficiency of MDLC composites with other conductive composites. The conductive composites with different architectures were classified into four types (Fig. 5f): continuous networks with randomly distributed sheets (type 1), vertically aligned structures with isotropic arrangement in horizontal direction (type 2), CNTs arrays (type 3), and two-dimensional sheet arrays (type 4). Different from above four types of conductive architectures, MDLC composites revealed significant enhancement efficiency in improving biaxial *λ* and *σ* (Fig. 5f and Table S2). The specific λ and σ in **n** direction reached 466 W m^−1^ K^−1^/(g cm^−3^) and 4.1 × 10^5^ S m^−1^/(g cm^−3^), respectively, indicating the highest enhancement efficiency. Such excellent biaxial conductivity demonstrates the merit of boundary-free sheargraphy method to precisely control the two-dimensional sheet ordering and potential usage of the skeleton as efficient thermal interface materials.

### Controllable Topological Structure and Programmable Polydomain Texture

**Fig. 6:**
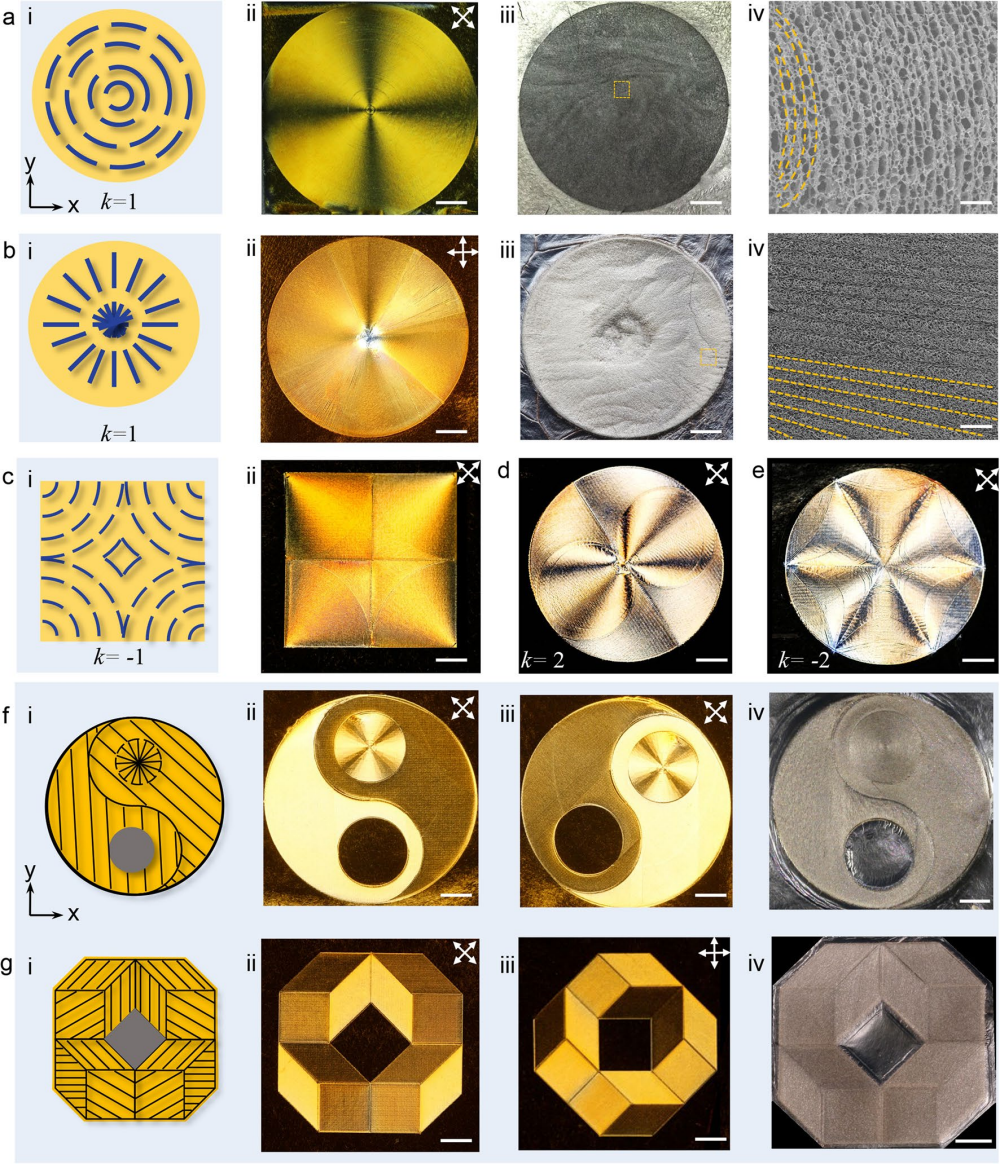
Topological configuration: **a** concentric-circle structure, **b** hedgehog topology with topological intensity of + 1, **c** the structure with topological intensity of − 1, **d** the structure with topological intensity of 2, and **e** the structure with topological intensity of -2. In each panel: (i) sheet arrangement profile, (ii) POM image, (iii) dried skeletons, and (iv) SEM images. Controllable polydomain: **f** Tai Chi pattern, **g** cell of quasi-crystalline with 8 rotational symmetry. In each panel: (i) sheet arrangement profile, (ii-iii) polarized texture before and after clockwise rotating the sample by 45°, (iv) dried skeletons. All images were from top view. Scale bars: a − c (iv) 200 µm; others 1 cm

Benefiting from arbitrariness and high precision of boundary-free sheargraphy method, we also achieved the free design of two-dimensional liquid crystals from MDLCs (without defect) to topological structures with tunable defect strength. By controllably introducing singularity into the MDLCs, diverse topological structures were fabricated (Fig. 6a–e). According to the sheet arrangement profile (Fig. 6a–b), schlieren textures with the concentric-circle structure and hedgehog defects [37] were prepared, presenting topological defect strength of + 1, while topological intensity of the structure in Fig. 6c was − 1. Topological structures with defect strength of + 2 and − 2 were also fabricated (Fig. 6d–e), indicating the free design of boundary-free sheargraphy for defect engineering. Optical images and SEM images (Fig. 6a–b (iii-iv)) jointly demonstrated that the GO sheets were aligned along sheet arrangement profile. In addition, complex polydomain were also designed by cohering MDLCs and disclinations (Figs. 6f and g (i-iii) and S12). Due to high ordering of the sub-domains, polydomain structures presented sensitive polarization responses and complex textures, such as Tai Chi, rotational repeating cell, and hexagonal rhombuses (Fig. S12). After freeze casting, the polydomain skeletons also exhibited the same sheet arrangement with their colloidal state (Fig. 6f and g (iv)). Controllably designing topological structures thus may provide a new route to tailor higher-order defects of two-dimensional colloids and design new optical components [38, 39].

Shear-induced alignment of anisotropy mesogens has been extensively used, such as blade coating, mechanical stirring, and spinning. These common methods merely result in a limited manipulation of sheet ordering due to intrinsic boundary layers. Our boundary-free sheargraphy breaks down the boundary layer dilemma and extends our limit to precisely fabricate monodomain liquid crystals in arbitrary macroscopic size. The achieved MDLCs exhibit centimeter-scale continuity and regularity. Compared with other patterning methods, such as photoalignment [40, 41], electromagnetic fields [7, 13], and freeze casting [34, 35], our method shows merits of high accuracy and simple equipment request, arbitrariness, and wide compatibility for two-dimensional colloids. High accuracy (2 µm) of boundary-free shearing fields, lower than the size of single GO sheet (35 µm), allows the construction of monodomain textures with high sheet ordering. High arbitrariness of boundary-free sheargraphy steers the rational design of higher-order topological defects and complex polydomain in a macroscopic three-dimensional space. This methodology is designable to precisely fabricate delicate topology of two-dimensional colloids and assemble solid metamaterials with desired functionalities.

## Conclusions

We achieved MDLCs of two-dimensional sheets in a free state through boundary-free sheargraphy. The cumulative shearing fields of 2 µm exhibited a uniform shear stress distribution, eliminating the borned *π* wall defects and intrinsic topological defects of liquid crystals. MDLCs exhibited the same polarization response and rheological anisotropy, compared with common polycrystalline GO liquid crystals. The bidirectionally ordered alignment of GO sheets promised a regular skeleton for the composites with distinctive conductivity. The bidirectionally specific *λ* and *σ* of MDLC/PDMS composites exceeded that of most previous nanocomposites, implying the potential usage of the MDLCs as lightweight thermal interface materials. Furthermore, we extended the topology design of two-dimensional colloids by controllably introducing singularities within MDLCs. Textures with defect strength from − 2 to + 2 and complex polydomain were realized. Our work is a new regime to study the structural order of soft matter and fabricate the metamaterials with tunable and highly anisotropic architectures.

### Supplementary Information

Below is the link to the electronic supplementary material.

Supplementary file1 (DOCX 5372 KB)
