# Correction: Monodomain Liquid Crystals of Two-Dimensional Sheets by Boundary-Free Sheargraphy

**DOI:** 10.1007/s40820-023-01173-8

**Published:** 2023-10-13

**Authors:** Min Cao, Senping Liu, Qingli Zhu, Ya Wang, Jingyu Ma, Zeshen Li, Dan Chang, Enhui Zhu, Xin Ming, Florian Puchtler, Josef Breu, Ziliang Wu, Yingjun Liu, Yanqiu Jiang, Zhen Xu, Chao Gao

**Affiliations:** 1 MOE Key Laboratory of Macromolecular Synthesis and Functionalization, Department of Polymer Science and Engineering, Key Laboratory of Adsorption and Separation Materials and Technologies of Zhejiang Province, Zhejiang University, 38 Zheda Road, Hangzhou, 310027 People’s Republic of China; 2 Shanxi-Zheda Institute of Advanced Materials and Chemical Engineering, Taiyuan, People’s Republic of China; 3 State Key Lab of Chemical Engineering, College of Chemical and Biological Engineering, Zhejiang University, 38 Zheda Road, Hangzhou, 310027 People’s Republic of China; 4 Bavarian Polymer Institute and Department of Chemistry, University of Bayreuth, Universitätsstrasse 30, 95440 Bayreuth, Germany

**Correction to: Nano-Micro Letters (2022) 14:192** https://doi.org/10.1007/s40820-022-00925-2

In this article Florian Puchtler at affiliation ‘University of Bayreuth’, Josef Breu at affiliation ‘University of Bayreuth’, and Ziliang Wu at affiliation ‘Zhejiang University’ was missing from the author Min Cao, Senping Liu, Qingli Zhu, Ya Wang, Jingyu Ma, Zeshen Li, Dan Chang, Enhui Zhu, Xin Ming, Florian Puchtler, Josef Breu, Ziliang Wu, Yingjun Liu, Yanqiu Jiang, Zhen Xu, Chao Gao list.

The original article has been corrected.

