# Supplementary material for: Monodomain Liquid Crystals of Two-Dimensional Sheets by Boundary-Free Sheargraphy
Source: Nanomicro Lett. 2022 Sep 19;14:192. doi: 10.1007/s40820-022-00925-2 (PMC9485412; doi:10.1007/s40820-022-00925-2)
Supplement: Supplementary file 1 — Supplementary file1 (DOCX 5372 KB) [file 40820_2022_925_MOESM1_ESM.docx]

Supporting Information for

**Monodomain Liquid Crystals of Two-Dimensional Sheets by** **Boundary-Free Sheargraphy**

Min Cao^1^, Senping Liu^1^, Qingli Zhu^1^, Ya Wang^1^, Jingyu Ma^1^, Zeshen Li^1^, Dan Chang^1^, Enhui Zhu^1^, Xin Ming^1^, Yingjun Liu^1, 2,^ *, Yanqiu Jiang^1, 3,^ *, Zhen Xu^1,^ *, Chao Gao^1,^ *

^1^MOE Key Laboratory of Macromolecular Synthesis and Functionalization, Department of Polymer Science and Engineering, Key Laboratory of Adsorption and Separation Materials & Technologies of Zhejiang Province, Zhejiang University, 38 Zheda Road, Hangzhou 310027, P. R. China

^2^Shanxi-Zheda Institute of Advanced Materials and Chemical Engineering, Taiyuan, P. R. China

^3^State Key Lab of Chemical Engineering, College of Chemical and Biological Engineering, Zhejiang University, 38 Zheda Road, Hangzhou 310027, P. R. China

^*^Corresponding authors. E-mail: [yingjunliu@zju.edu.cn](mailto:yingjunliu@zju.edu.cn) (Yingjun Liu); [jiangyanqiu@zju.edu.cn](mailto:jiangyanqiu@zju.edu.cn) (Yanqiu Jiang); [zhenxu@zju.edu.cn](mailto:zhenxu@zju.edu.cn) (Zhen Xu); [chaogao@zju.edu.cn](mailto:chaogao@zju.edu.cn) (Chao Gao)

**S1 Supplementary Figures**


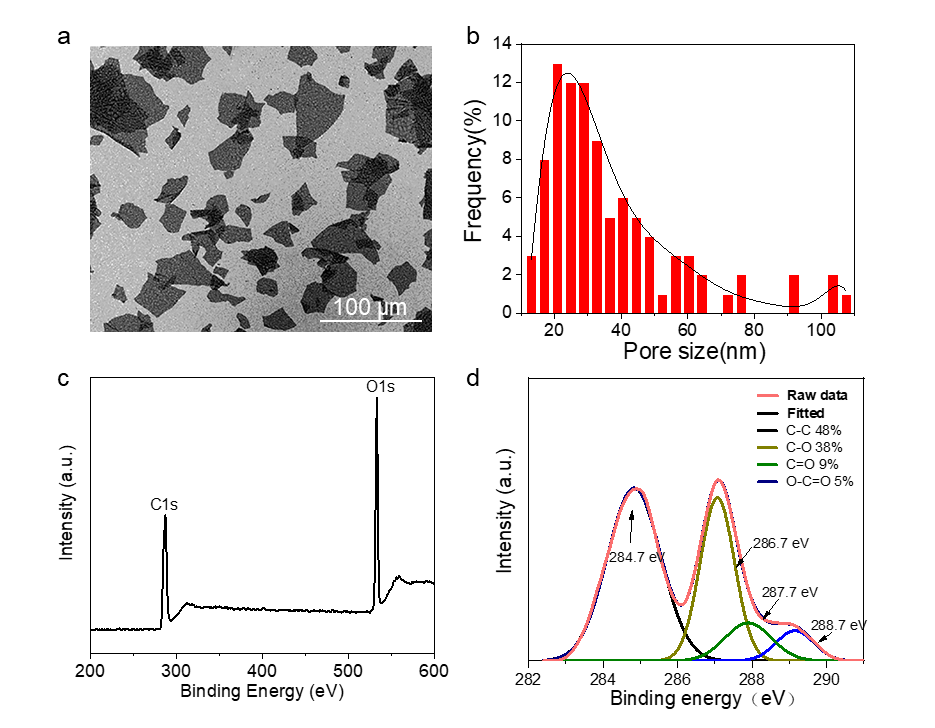


**Fig. S1** Characterization of GO sheets. (**a**) SEM images and (**b**) size distribution of GO sheets. The size distribution of GO sheets was about 20-50 µm. (**c**) XPS spectra of GO and (**d**) C 1*s* spectrum. The corresponding C/O ratio was 2.1


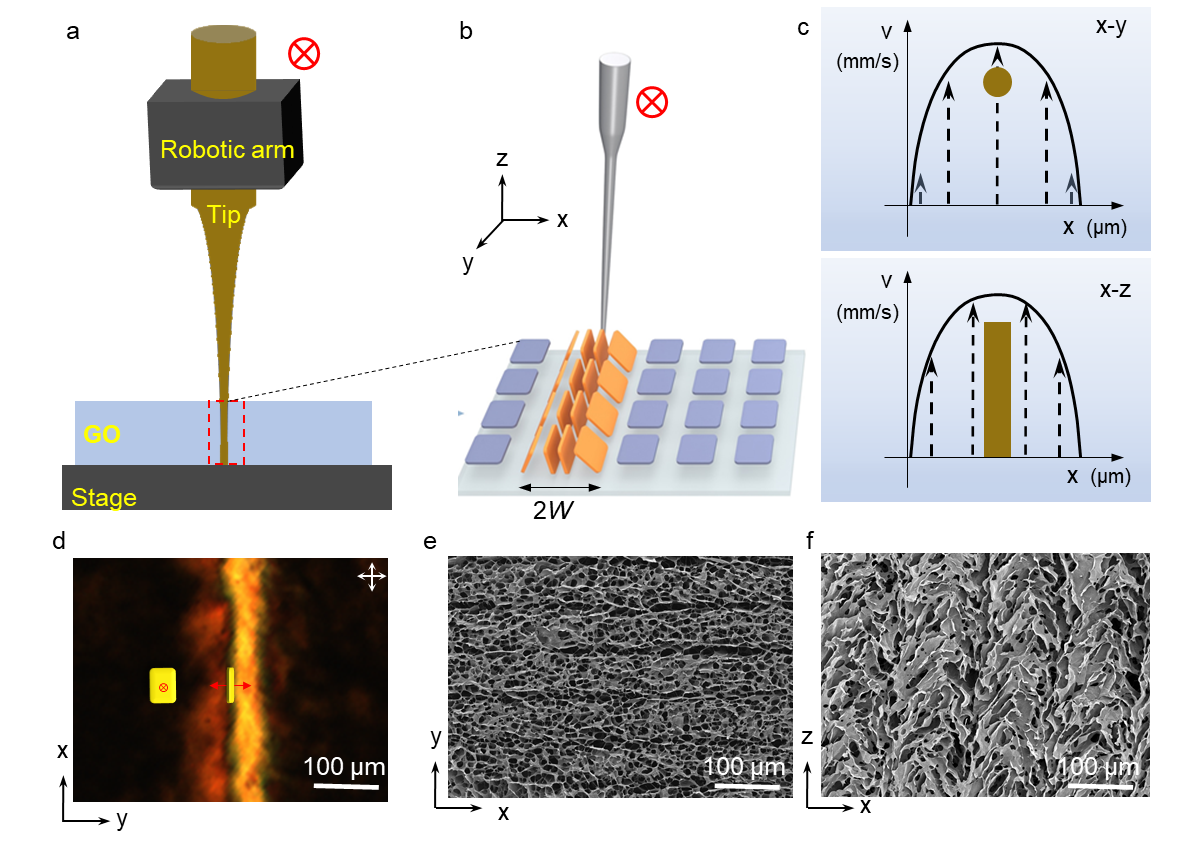


**Fig. S2** (**a-b**) Schematic of boundary-free sheargraphy method. (**c**) Velocity distribution of shearing filed in both x-y and x-z plane, respectively. (**d**) A π wall upon single shearing. (**e-f**) SEM images of π wall from both x-y and x-z plane, respectively


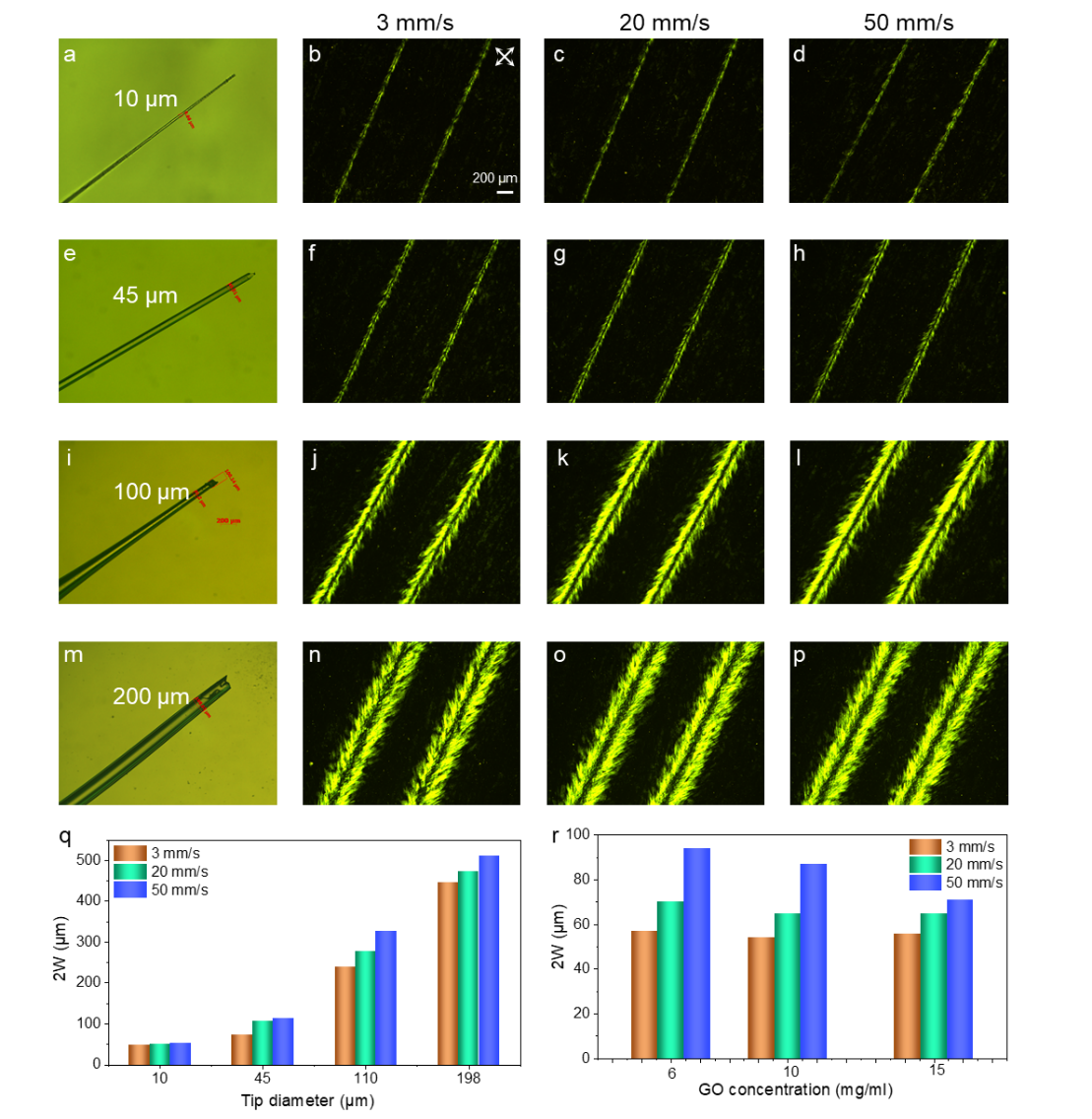


**Fig. S3** Images of tips with diameters around (**a**) 10 μm, (**e**) 45 μm, (**i**) 100 μm, and (**m**) 200 μm respectively. POM images of π wall induced by tip with diameter of (**b-d**) 10 μm, (**f-h**) 45 μm, (**j-l**) 100 μm, and (**n-p**) 200 μm at shearing speed of 3, 20, and 50 mm s^-1^, respectively. (**q**) Width (2*W*) of π wall tuned from approximately 48 μm to 510 μm on demand. (**r**) The influence of GO concentration on 2*W*. 2*W* from 50 to 90 μm gradually decreased to the range of 50 to 70 μm as viscosity of GO improved. Scale bar: 200 µm


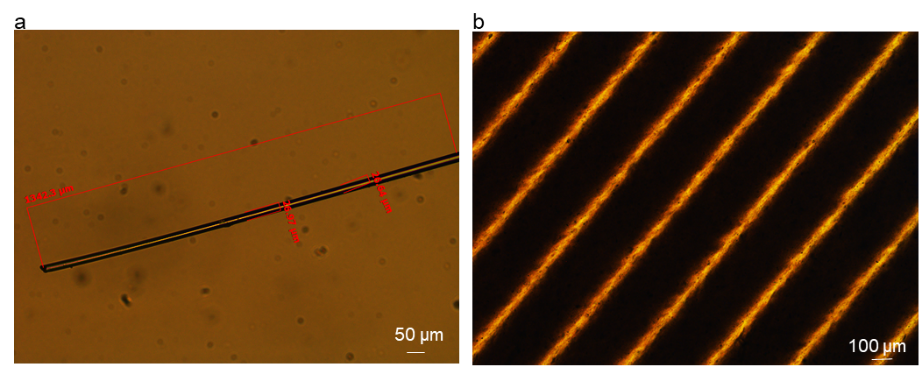


**Fig. S4 (a)** Optical image of the tip with a diameter of around 26 μm. (**b**) POM images of π-wall with a width of 50 μm.

**Table S1** Processing parameters of boundary-free sheargraphy

| ***W* (µm)** | 25 | 25 | 25 | 25 | 25 |
| --- | --- | --- | --- | --- | --- |
| ***D* (µm)** | 200 | 50 | 25 | 10 | 2 |
| ***D/W*** | 8 | 2 | 1 | 0.4 | 0.08 |

**
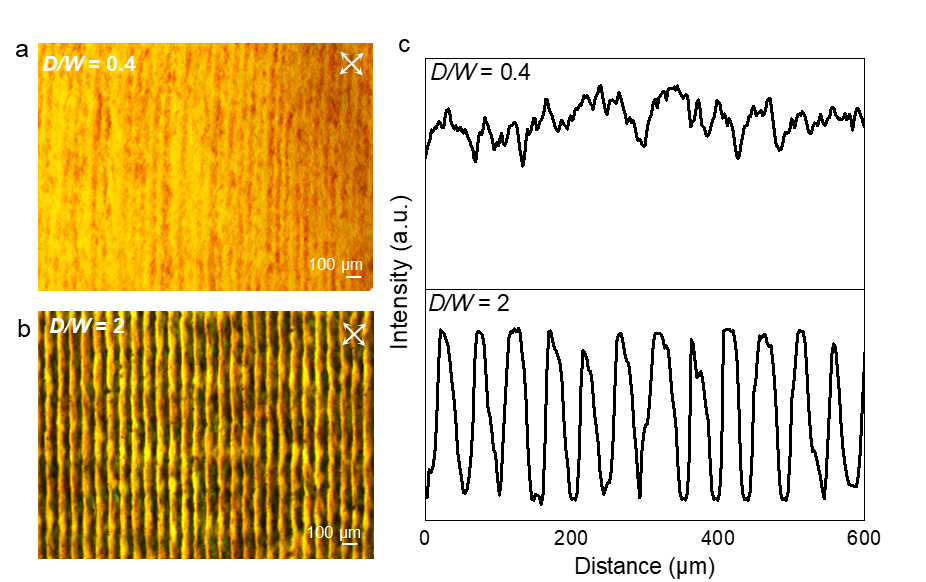
**

**Fig. S5** (**a**) POM images of sample with *D/W* of 0.4. (**b**) POM images of sample with *D/W* of 2. (**c**) Transmitted intensity of POM images


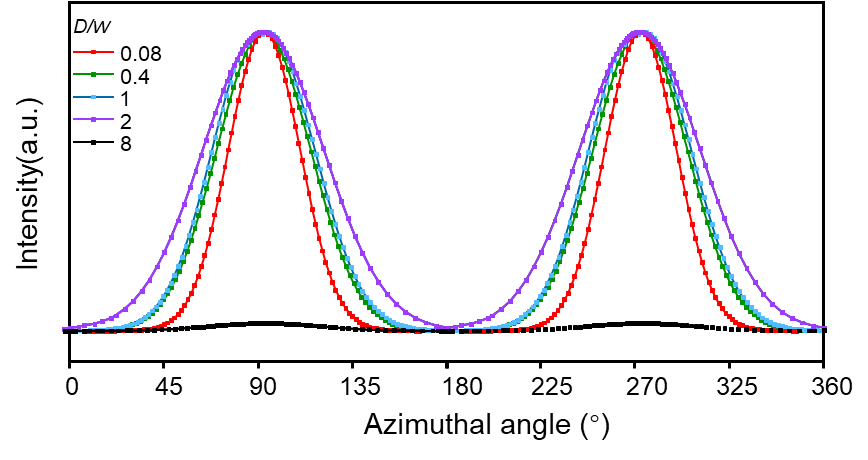


**Fig. S6** The azimuthal angle plot of SAXS pattern with the increasing *D/W*


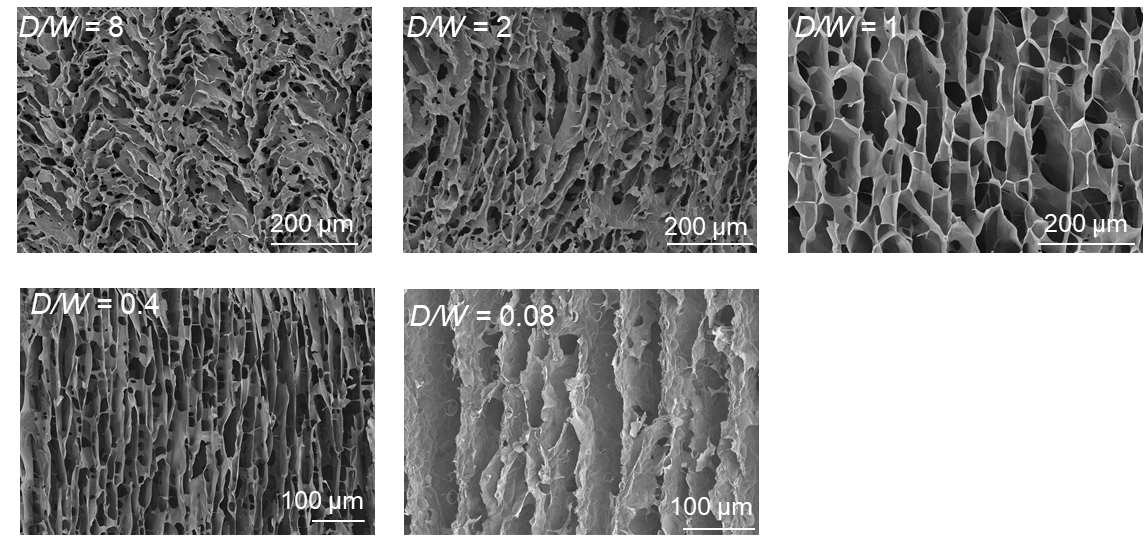


**Fig. S7** Cross-sectional SEM images with changing *D/W*. After being quenched in liquid nitrogen and freeze drying, these frozen configurations distinctively revealed that GO sheets were vertically aligned when *D/W* was controlled at 0.08

**Note S1 Transmitted brightness variation of linearly polarized light through MDLCs and common GO LCs as the angle changes.**

In general, the incident linearly polarized light is converted to elliptically polarized light, with a component that can pass through the crossed analyzer; the sample appears bright. The transmitted intensity is given by Eq. (S1):

$I=I_{0}\cdot\sin^{2} 2\varphi\cdot\sin^{2} \frac{\delta}{2}$ (S1)

where $I_{0}$ is the light intensity after the polarizer and $\varphi$ is the azimuthal angles, *i.e.* the angle between the analyzer and the projection of the optic axis onto the sample plane. The second term$(\sin^{2} \frac{\delta}{2})$ is responsible for the beautiful colors of the liquid crystalline textures, which relies on the thickness, aspect ratio, wavelength, and concentration. In our experiments, the parameters of the POM test are the same and the samples have the same GO concentration, thickness, *etc.*, thus transmitted intensity is only the function of azimuthal angle, *i.e.* $I\propto\sin^{2} 2\varphi$, which has a maximum value for$\varphi$= 45° and a minimum for $\varphi$ = 0° and $\varphi$= 90°.

In Fig. 2c, the transmitted brightness of the MDLCs displayed a maximum transmitted intensity for$\varphi$= 45°, changing periodically with a bright-dark transition per rotated 90°. The polarization response of MDLCs was consistent with Equation 1, implying that the angle between the directors of GO sheets and the cross polarizer was 45°. In contrast, conventional GO LCs had a lower transmitted intensity because of the polydomain feature. These results above indicated GO sheets of MDLCs were both vertically aligned and aligned along the shearing direction.

**Fig. S8** Anisotropic flow properties of common GO LCs in orthogonal directions


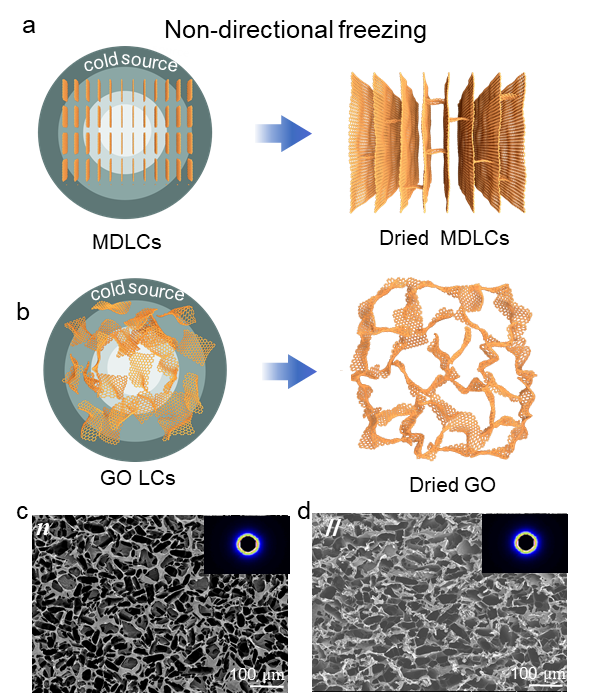


**Fig. S9** (**a**) Scheme of non-directional freezing technique and resulting skeletons of MDLCs. The color variation represents the temperature gradient was isotropic when freeze casting. (**b**) Corresponding dried GO skeletons by using non-directional freezing technique. (**c-d**) SEM images of common GO skeletons from *n* and*Ⅱ*directions, and the insert are SAXS patterns

In contrast, the skeleton of common GO LCs was random and isotropic both from *n* and*Ⅱ*directions (Fig. S7c, d). The inserted SAXS images of the skeleton showed isotropic scattering rings, indicating the disorder sheet arrangement.


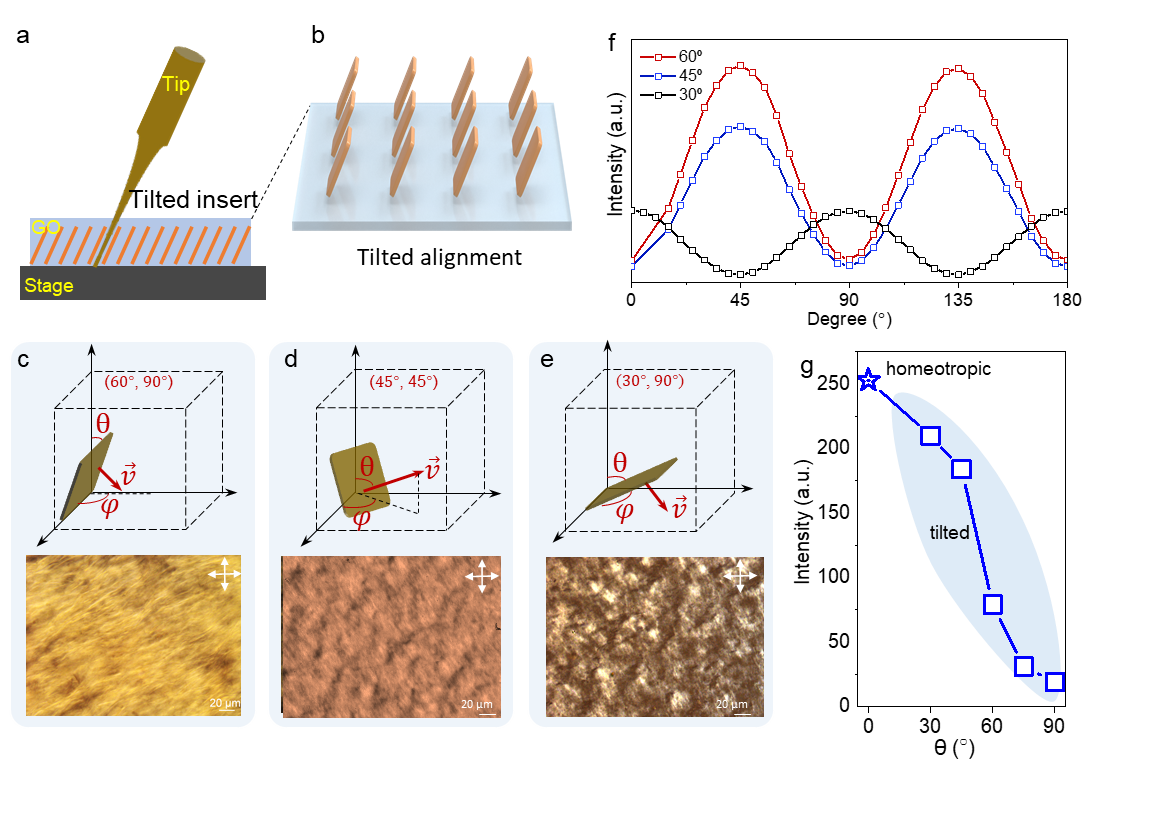


**Fig. S10** (**a-b**) Control mechanism of spatial angle of the shearing field. (**c-e**) Corresponding textures of MDLCs with tilted alignment (θ, 𝜑): (60°, 90°), (45°, 45°), (30°, 90°). (**f**) Transmitted intensity of tilted MDLCs in a period of 180°. (**g**) The maximum transmitted intensity of MDLCs with varying tilted sheet orientation

The spatial angle of the shearing field can be easily controlled by adjusting the spatial angle between stage and immersed tip. When tip was inserted into the liquid crystal at a titled angle (Fig. S10a, b), tilted shearing fields were generated, inducing GO sheets to reorient along the tilted shearing fields. After cumulative shearing, GO sheets were in tilted alignment following tip movement. The tilted MDLCs exhibited uniform texture and sensitive polarization response (Fig. S10c-f). Due to the tilted arrangement of GO sheets, the transmitted light intensity gradually decreased as the θ increased (Fig. S10e).

**
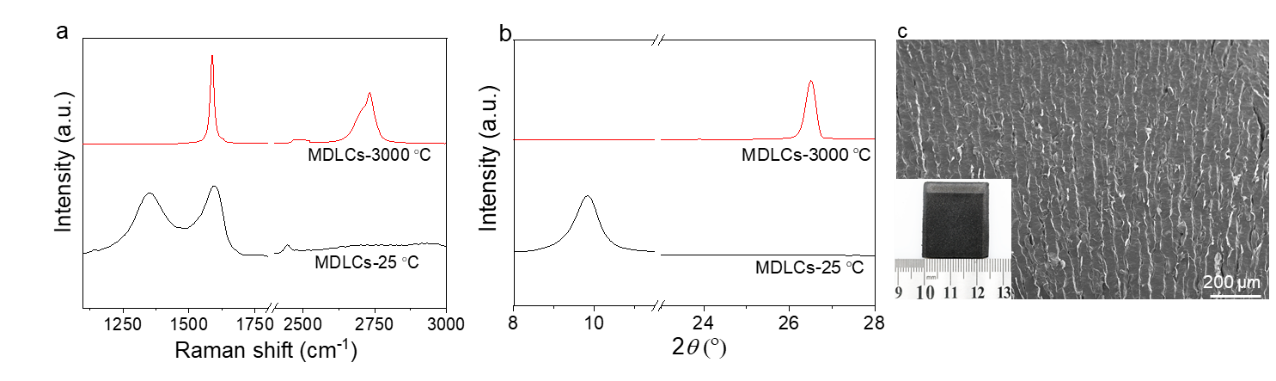
**

**Fig. S11** (**a**) XRD and (**b**) Raman spectrum of skeletons of MDLCs before and after thermal graphitization. (**c**) Cross-sectional SEM image of the MDLC/PDMS composites. Insert was the optical image

XRD and Raman results implied high crystalline and perfect graphene structure after thermal graphitization (Fig. S11a, b). The regular skeleton of MDLCs was well reserved after PDMS infiltration, showing the long-range vertically-aligned lamellar structure (Fig. S11c).

**Table S**2 Comparison of specific thermal conductivity and specific electric conductivity between our work and other polymer composites

| **Type** | **filler** | **Density**  **(g/cm^3^)** | **Specific σ**  **(S m^-1^/ (g cm^-3^))** | **Specific λ**  **(W m^-1^ K^-1^/(g cm^-3^))** | **Refs.** |
| --- | --- | --- | --- | --- | --- |
| 1 | CNT foam | 0.26 | 3.97 | 4.30 | [S2] |
|  |  | 0.312 | 0.94 | 5.56 |  |
|  |  | 0.408 | 0.38 | 7.69 |  |
|  |  | 0.69 | 0.26 | 9.62 |  |
|  |  | 1.21 | 0.17 | 10.54 |  |
|  | MXene | 0.25 | - | 30800 | [S3] |
|  |  | 0.18 | - | 65000 | [S4] |
|  |  | 0.0089 | 8.7 | 449 | [S5] |
|  | Graphene nanoplates  & CNTs | 0.07945 | 12.59 | 7.55 | [S6] |
|  |  | 0.0908 | 330.40 | 8.81 |  |
|  |  | 0.1135 | 352.42 | 9.69 |  |
| 2 | Graphene | 0.37 | 89.19 | 22.78 | [S7] |
|  |  | 0.385 | 28000 | 92.21 |  |
|  | Graphene nanoplates | 0.1135 | 0.88 | 10.6 | [S8] |
|  |  | 0.227 | 48.46 | 11.0 |  |
|  |  | 0.3405 | 58.74 | 14.7 |  |
|  |  | 0.454 | 132.16 | 15.4 |  |
|  |  | 0.5675 | 123.37 | 17.6 |  |
| 3 | CNTs | 0.06 | 13800 | 14 | [S9] |
|  |  | 0.06 | 23000 | 20 |  |
|  |  | 0.168 | 7142 | 89 | [S10] |
|  |  | 0.168 | 7142 | 136 | [S11] |
| 4 | Graphene | 0.0059 | 118644.07 | 423.73 | [S12] |
|  |  | 0.00944 | 105932.20 | 349.58 |  |
|  |  | 0.0142 | 71830.99 | 338.03 |  |
|  |  | 0.0177 | 59548.02 | 371.19 |  |
|  | Graphene | 0.017 | 58823.53 | 58.82 | [S13] |
|  |  | 0.044 | 47727.27 | 136.36 |  |
|  |  | 0.077 | 38961.04 | 97.40 |  |
| MDLC  ***Ⅱ*** | Graphene | 0.0086 | 143 | 81395 | ThisWork |
|  |  | 0.01 | 228 | 100000 |  |
|  |  | 0.0122 | 319 | 83606 |  |
|  |  | 0.0145 | 321 | 72191 |  |
| MDLC  ***n*** |  | 0.0086 | 215 | 52325 |  |
|  |  | 0.01 | 348 | 222412 |  |
|  |  | 0.0122 | 425 | 338000 |  |
|  |  | 0.0145 | 465 | 408479 |  |

The filler/polymer nanocomposites based on weight fractions can be converted to volume fractions using Eq. (S2):

$V_{f}=\frac{\rho_{m}W_{f}}{{\rho_{m}W_{f}+(1-W_{f})\rho}_{f}}$ (S2)

where *m* and *f* are the matrix and filler. *ρ* and *W* represent the density and weight fractions, respectively. In our investigation, the density values of rubber, epoxy, graphene, CNTs, and Mxene were taken to be 1.01, 1.17, 2.27, 2.1, and 5.2 g cm^-3^, respectively [7, 14, 15]. The density of the conductive architectures in nanocomposites based on volume fractions can be calculated using Eq. (S3):

$\rho_{true}=V_{f}{ⅹ\rho}_{f}$ (S3)

where $\rho_{true}$ is true density of conductive filler skeleton in nanocomposites. $V_{f}$ is volume fractions and $\rho_{f}$is the filler bulk density.


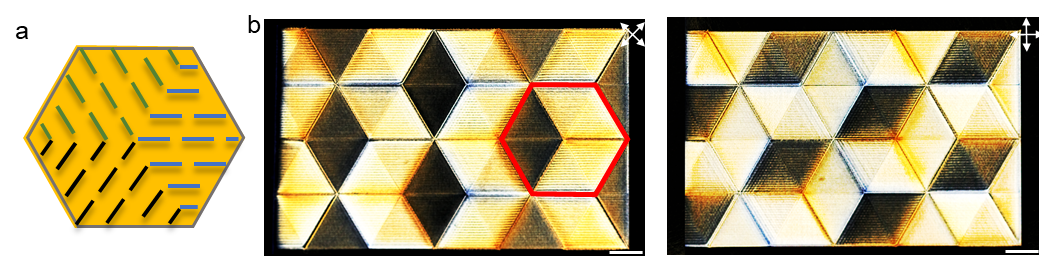


**Fig. S12** (**a**) Sheet arrangement profile of GO sheets. (**b-c**) Textures of hexagonal rhombuses with GO sheets parallel to short diagonals. Scale bar: 1 cm

**Supplementary References**

1. Y.Q. Jiang, F. Guo, Z. Xu, W.W. Gao, C. Gao, Artificial colloidal liquid metacrystals by shearing microlithography. Nat. Commun. **10**, 4111 (2019). <http://doi.org/10.1038/s41467-019-11941-z>
2. F. Zhang, Y.Y. Feng, M.M. Qin, L. Gao, Z.Y. Li et al., Stress controllability in thermal and electrical conductivity of 3D elastic graphene‐crosslinked carbon nanotube sponge/polyimide nanocomposite. Adv. Func. Mater. **29**(25), 1901383 (2019). <http://doi.org/10.1002/adfm.201901383>
3. P. Zhang, R.A. Soomro, Z.R.X. Guan, N. Sun, B. Xu, 3D carbon-coated MXene architectures with high and ultrafast lithium/sodium-ion storage. Ener. Stor. Mater. **29**(1), 163-171 (2020). [https://doi.org/10.1016/j.ensm.2020.04.016](http://doi.org/https://doi.org/10.1016/j.ensm.2020.04.016)
4. Y.Z. Li, X.T. Zhang, Electrically conductive, optically responsive, and highly orientated Ti_3_C_2_T_x_ MXene aerogel fibers. Adv. Func. Mater. **32**(4), 2107767 (2022). [https://doi.org/10.1002/adfm.202107767](http://doi.org/https://doi.org/10.1002/adfm.202107767)
5. J. Liu, H.B. Zhang, X. Xie, R. Yang, Z. Liu et al., Multifunctional, superelastic, and lightweight MXene/polyimide aerogels. Small **14**(45), 1802479 (2018). <http://doi.org/10.1002/smll.201802479>
6. K. Wu, C.X. Lei, R. Huang, W.X. Yang, S.G. Chai et al. Design and preparation of a unique segregated double network with excellent thermal conductive property. ACS Appl. Mater. Interfaces **9**(8), 7637-7647 (2017). <http://doi.org/10.1021/acsami.6b16586>
7. F. An, X.F. Li, P. Min, P.F. Liu, Z.G. Jiang et al., Vertically aligned high-quality graphene foams for anisotropically conductive polymer composites with ultrahigh through-plane thermal conductivities. ACS Appl. Mater. Interfaces **10**(20), 17383-17392 (2018). <http://doi.org/10.1021/acsami.8b04230>
8. H. Jung, S. Yu, N.S. Bae, S.M. Cho, R.H. Kim et al., High through-plane thermal conduction of graphene nanoflake filled polymer composites melt-processed in an L-shape kinked tube. ACS Appl. Mater. Interfaces **7**(28), 15256-15262 (2015). <http://doi.org/10.1021/acsami.5b02681>
9. M.B. Jakubinek, M.A. White, G. Li, C. Jayasinghe, W. Cho et al., Thermal and electrical conductivity of tall, vertically aligned carbon nanotube arrays. Carbon **48**(13), 3947-3952 (2010). <http://doi.org/10.1016/j.carbon.2010.06.063>
10. N. Matsumoto, A. Oshima, S. Sakurai, M. Yumura, K. Hata et al., Scalability of the heat and current treatment on swcnts to improve their crystallinity and thermal and electrical conductivities. Nanoscale Res. Lett. **10**(1), 220-220 (2015). <http://doi.org/10.1186/s11671-015-0917-0>
11. I.N. Ivanov, A.A. Puretzky, G. Eres, H.P. Wang, Z.W. Pan et al., Fast and highly anisotropic thermal transport through vertically aligned carbon nanotube arrays. Appl. Phys. Lett. **89**(22), 223110 (2006). <http://doi.org/10.1063/1.2397008>
12. X. Li, P. Liu, X. Li, F. An, P. Min et al., Vertically aligned, ultralight and highly compressive all-graphitized graphene aerogels for highly thermally conductive polymer composites. Carbon **140**(12), 624-633 (2018). <http://doi.org/10.1016/j.carbon.2018.09.016>
13. H. Fang, H. Guo, Y. Hu, Y. Ren, P. Hsu et al., In-situ grown hollow Fe_3_O_4_ onto graphene foam nanocomposites with high EMI shielding effectiveness and thermal conductivity. Compos. Sci. Technol. **188**(1), 107975 (2020). <http://doi.org/10.1016/j.compscitech.2019.107975>
14. Y. Li, F. Xu, Z. Lin, X. Sun, Q. Peng et al., Electrically and thermally conductive underwater acoustically absorptive graphene/rubber nanocomposites for multifunctional applications. Nanoscale **9**(38), 14476-14485 (2017). <http://doi.org/10.1039/c7nr05189a>
15. J.S. Lewis, Z. Barani, A.S. Magana, F. Kargar, A.A. Balandin, Thermal and electrical conductivity control in hybrid composites with graphene and boron nitride fillers. Mater. Res. Exp. **6**(8), 85325-85333, (2019). <http://doi.org/10.1088/2053-1591/ab2215>
